# Clinical Prediction Models for Pneumonia in Children Presenting to an Emergency Department in a Resource-Limited Setting Using Lung Ultrasound Diagnosis as the Gold Standard

**DOI:** 10.7759/cureus.81360

**Published:** 2025-03-28

**Authors:** Darlene R House, Yogendra Amatya, Benjamin K Nti, Frances M Russell

**Affiliations:** 1 Emergency Medicine, Icahn School of Medicine at Mount Sinai, New York, USA; 2 General Practice and Emergency Department, Patan Academy of Health Sciences, Kathmandu, NPL; 3 Emergency Medicine, Indiana University School of Medicine, Indianapolis, USA

**Keywords:** developing countries, diagnosis, lung ultrasound, pediatrics, pneumonia

## Abstract

Introduction: Clinical prediction rules for pediatric pneumonia often rely on radiographic pneumonia for diagnosis; however, lung ultrasound has higher diagnostic accuracy. Our objective was to derive a clinical prediction model for pneumonia in children under five using lung ultrasound as the criterion standard.

Methods: This was a prospective study of children under five presenting to an emergency department (ED) with respiratory complaints in a resource-limited setting. Clinical findings, chest X-ray, and lung ultrasound results were recorded for each patient. Classification tree models were used to predict pneumonia using lung ultrasound as the criterion standard. Separate models were used without and with inclusion of chest X-ray results.

Results: Of 386 patients enrolled, 125 patients (32.4%) had pneumonia on lung ultrasound. The mean age was 20.8 (SD 15.5) months. Using recursive feature selection, three variables provided the best prediction for pneumonia, namely, crepitations, retractions, and difficulty breathing, demonstrating a sensitivity of 74.2% and specificity of 38.5%. The algorithm including chest X-ray provided a sensitivity of 51.6% and specificity of 87.7%.

Conclusions: Using lung ultrasound as the gold standard, no single clinical finding or combination of clinical findings provided enough accuracy to reliably diagnose pneumonia in children under five years.

## Introduction

Pneumonia is a leading cause of death in children under five years of age worldwide [1]. These deaths may be prevented by early detection and targeted antibiotic therapy [2]. However, the diagnosis is not always clear on presentation to healthcare facilities. Missed diagnosis may lead to increased morbidity and mortality, while overdiagnosis may lead to unnecessary antibiotic use, which may further lead to adverse outcomes such as increased antibiotic resistance, allergic reactions, and unnecessary costs for patients [3]. Therefore, there is a critical need to use clinical tools and diagnostic capabilities to better improve diagnosis and management.

Many studies have evaluated clinical signs and symptoms that may predict pneumonia [4-8]. Studies have found that there is no single physical exam finding that predicts pneumonia [6,9]. The World Health Organization (WHO) criteria diagnose pneumonia based on tachypnea; however, tachypnea has not been shown to strongly predict pneumonia [10,11]. Studies evaluating clinical predictors have been based on admission diagnoses or chest X-rays, which have limited sensitivity and specificity for the diagnosis of pneumonia [7-9,12,13]. Lung ultrasound has been shown to be more sensitive and specific for the diagnosis of pneumonia in both children and adults compared to chest X-ray [14-18]. Specifically, in pediatric pneumonia, multiple meta-analyses have shown lung ultrasound to have a collective sensitivity of 93-95% [18-20]. Currently, no studies have evaluated clinical predictors using lung ultrasound for the diagnosis of pneumonia in children.

The objective of this study was to develop a prediction model for the diagnosis of pneumonia in children under five presenting to an emergency department (ED) in a resource-limited setting using lung ultrasound as the criterion standard.

## Materials and methods

Study design

This was a prospective observational cross-sectional study of pediatric patients presenting with fever or respiratory complaints to the emergency department at Patan Hospital in Lalitpur, Nepal, from June 1, 2018 to March 20, 2020. Ethical approval was obtained from the Patan Institutional Review Committee (reference number: drs1805271178). The study was performed as part of our registered study with clinicaltrials.gov (registration number NCT03630380).

Study setting and population

Patan Hospital is a large urban hospital with a 35-bed emergency department. The emergency department has an annual volume of approximately 48,000 patients, including approximately 8,000 pediatric visits. The overall admission rate is 20%. Patients presenting under five years of age with fever or respiratory complaints were included. Parents were consented for inclusion of the child in the study. 

Study protocol

Data were collected on pediatric patients meeting the above inclusion criteria. Data collected included demographics (age, gender), symptoms (presence or absence of fever, cough, difficulty breathing), duration of symptoms, vital signs (temperature, respiratory rate, oxygen saturation), and physical exam findings (retractions or indrawing of chest, crepitations, or wheezing). If the child only had a fever, but all other vitals and physical exam findings were normal, the exam was documented as normal. Data were entered on a standardized data collection form.

After clinical evaluation, a bedside lung ultrasound was performed by a clinician trained to perform lung ultrasound. Training consisted of two hours of didactics and at least 20 previous lung ultrasound scans that were observed or reviewed. Lung ultrasound was provided free for patients. The clinician performing the bedside lung ultrasound was blinded to clinical information and results of any chest imaging. A Sonosite M Turbo (Fujifilm Sonosite, Inc.) ultrasound machine with a curvilinear probe was used. High-frequency linear probes were not available in this setting, similar to many low- and middle-income countries and rural settings; therefore, the curvilinear probe was used. In accordance with previous literature, the ultrasound examination included 10 views: two anterior views and two lateral views (one including the costophrenic angle), and one posterior view on each hemithorax [15,21,22]. The physician recorded ultrasound findings and interpretation directly after the ultrasound was complete. An ultrasound diagnosis of pneumonia was defined as the presence of unilateral B lines (>3) or subpleural lung consolidation. All ultrasounds were reviewed by an expert sonographer for the presence or absence of pneumonia. The expert reviewer was blinded to the clinician’s interpretation and patient data.

All patients had a single view posterior-anterior (PA) chest X-ray as a part of the standard evaluation. The WHO Chest Radiography in Epidemiological Studies (CRES) methodology was used to standardize interpretation and define radiographic pneumonia [23,24]. Chest X-rays were read by a board-certified radiologist, who was blinded to the clinical presentation and the results of any other imaging. Chest X-ray readings were recorded on the standardized data collection form.

Sample size calculation

Based on the need for at least 10 outcome events for each independent variable, 120 patients with pneumonia confirmed on lung ultrasound were needed for the study [25]. With an estimated 30% prevalence of pneumonia in children that present to Patan Hospital Emergency Department, a sample size of approximately 400 patients was calculated.

Statistical analysis

Descriptive statistics were used to determine prevalence of pneumonia in this patient population along with frequency of signs and symptoms in patients with pneumonia. Sensitivity, specificity, and likelihood ratios were calculated for each finding within clinical history, physical exam, and chest X-ray compared with lung ultrasound.

Classification tree models were used to predict pneumonia. Lung ultrasound was used as the criterion standard for the diagnosis of pneumonia. Separate models were used without and with inclusion of chest X-ray results. Data were randomly split into training (75%) and test (25%) samples. The data in the training samples were used to develop the models. The classification trees for the training sample were grown using 10-fold cross-validation, repeated five times to provide the variables that best predict pneumonia. The models were then applied to the test data set to calculate sensitivity, specificity, and F-score. Classification trees were analyzed using the rpart and rpart.plot packages of R statistical software (http://www.rstudio.com, https://www.R-project.org).

Kappa analysis was used to determine inter-rater reliability for interpretation of lung ultrasounds between the clinician and expert reviewer.

## Results

Three hundred eighty-six patients were included in the study. We ended the study before reaching 400 patients due to the COVID-19 pandemic. Of the 386 patients, 125 patients (32.4%) were diagnosed with pneumonia by lung ultrasound. The mean age was 20.8 (SD 15.5) months with 42% female patients (Table 1).

**Table 1:** Demographics

	Training		Testing		
	No pneumonia (N = 196)	Pneumonia (N = 94)	No pneumonia (N = 65)	Pneumonia (N = 31)	Total (N = 386)
Age (months)					
Mean (SD)	20.3 (15.1)	21.6 (16.3)	20.0 (13.2)	23.3 (19.5)	20.8 (15.5)
Range	0.2 - 59.0	0.2 - 59.0	0.5 - 58.0	2.0 - 59.0	0.2 - 59.0
Gender					
F	82 (41.8%)	37 (39.4%)	29 (44.6%)	14 (45.2%)	162 (42.0%)
M	114 (58.2%)	57 (60.6%)	36 (55.4%)	17 (54.8%)	224 (58.0%)

Diagnostic accuracy for each individual finding is summarized (Table 2). While symptoms of fever and cough had the highest sensitivity (87.2% and 96.0%, respectively), neither symptom was specific for pneumonia (16.5% and 11.2% respectively). Of the physical exam findings, tachypnea had the highest sensitivity at 67% (95% CI: 58-75%) while retractions and crepitations had the highest positive likelihood ratios. Chest X-ray demonstrated 68% sensitivity and 96% specificity (Table 2).

**Table 2:** Diagnostic accuracy of individual clinical findings and chest X-ray *The WHO definition of tachypnea was used.

	Sensitivity (95% CI)	Specificity	Positive likelihood ratio	Negative likelihood ratio
Cough	96.0% (91.0-98.7)	11.2% (7.6-15.6)	1.1 (1.0-1.1)	0.4 (0.1-0.9)
Fever	87.2% (80.0-92.5)	16.5% (12.2-21.6)	1.0 (1.0-1.1)	0.8 (0.5-1.3)
Difficulty breathing	67.2% (58.2-75.3)	58.6% (52.4-64.7)	1.6 (1.3-2.0)	0.6 (0.4-0.7)
Duration of Illness (>3d)	57.1% (48.0-65.9)	68.5% (62.4-74.1)	1.8 (1.4-2.3)	0.6 (0.5-0.8)
Oxygen Saturation (<92%)	44% (35.1-53.2)	76.9% (71.3-81.9)	1.9 (1.4-2.6)	0.7 (0.6-0.8)
Temperature (>38)	43.2% (34.4-52.4)	54.6% (48.4-60.8)	1.0 (0.8-1.2)	1.0 (0.9-1.3)
Tachypnea*	67.2% (58.2-75.3)	60.4% (54.2-66.4)	1.7 (1.4-2.1)	0.5 (0.4-0.7)
Retractions	34.4% (26.1-43.4)	86.9% (82.2-90.8)	2.6 (1.8-3.9)	0.8 (0.7-0.9)
Crepitations	47.2% (38.2-56.3)	82.3% (77.1-86.8)	2.7 (1.9-3.7)	0.6 (0.5-0.8)
Wheezing	22.4% (15.4-30.7)	78.9% (73.4-83.7)	1.1 (0.7-1.6)	1.0 (0.9-1.1)
Normal Exam	16.0% (10.1-23.6)	54.6% (48.4-60.8)	0.4 (0.2-0.5)	1.5 (1.3-1.8)
Chest X-ray	68.0% (59.1-76.1)	96.5% (93.5-98.4)	19.6 (10.2-37.8)	0.3 (0.3-0.4)

The distribution of clinical characteristics for the training and testing groups are summarized (Table 3). The training group for developing the model consisted of 94 patients with pneumonia and 196 patients without pneumonia while the testing group had 31 and 65 patients, respectively. The groups were similar in the distribution of clinical characteristics (Table 3).

**Table 3:** Clinical characteristics for the training and testing models

	Training		Testing		
	No pneumonia (N = 196)	Pneumonia (N = 94)	No pneumonia (N = 65)	Pneumonia (N = 31)	Total (N = 386)
Cough	175 (89.3%)	92 (97.9%)	57 (87.7%)	28 (90.3%)	352 (91.2%)
Illness duration (days)					
Mean (SD)	3.3 (2.5)	4.6 (2.7)	3.0 (1.8)	4.4 (3.0)	3.7 (2.6)
Range	1.0 - 15.0	1.0 - 15.0	1.0 - 8.0	1.0 - 14.0	1.0 - 15.0
Oxygen saturation					
Mean (SD)	94.9 (4.0)	91.3 (6.9)	94.3 (4.2)	93.8 (4.1)	93.8 (5.1)
Range	75.0 - 100.0	65.0 - 99.0	76.0 - 100.0	84.0 - 99.0	65.0 - 100.0
Temperature					
Mean (SD)	99.9 (2.2)	100.1 (2.0)	100.2 (2.0)	99.6 (1.9)	100.0 (2.1)
Range	91.2 - 105.1	96.5 - 106.0	96.6 - 104.2	96.0 - 103.0	91.2 - 106.0
Fever	163 (83.2%)	81 (86.2%)	55 (84.6%)	28 (90.3%)	327 (84.7%)
Tachypnea	75 (38.3%)	64 (68.1%)	29 (44.6%)	20 (64.5%)	188 (48.7%)
Respiratory rate					
Mean (SD)	40.7 (13.3)	48.5 (13.8)	42.5 (14.0)	48.3 (15.4)	43.5 (14.1)
Range	20.0 - 76.0	23.0 - 80.0	22.0 - 78.0	22.0 - 85.0	20.0 - 85.0
Difficulty breathing	76 (38.8%)	65 (69.1%)	32 (49.2%)	19 (61.3%)	192 (49.7%)
Retractions	21 (10.7%)	35 (37.2%)	13 (20.0%)	8 (25.8%)	77 (19.9%)
Crepitations	33 (16.8%)	48 (51.1%)	13 (20.0%)	11 (35.5%)	105 (27.2%)
Wheezing	40 (20.4%)	24 (25.5%)	15 (23.1%)	4 (12.9%)	83 (21.5%)
Normal	87 (44.4%)	14 (14.9%)	31 (47.7%)	6 (19.4%)	138 (35.8%)

**Figure 1:**
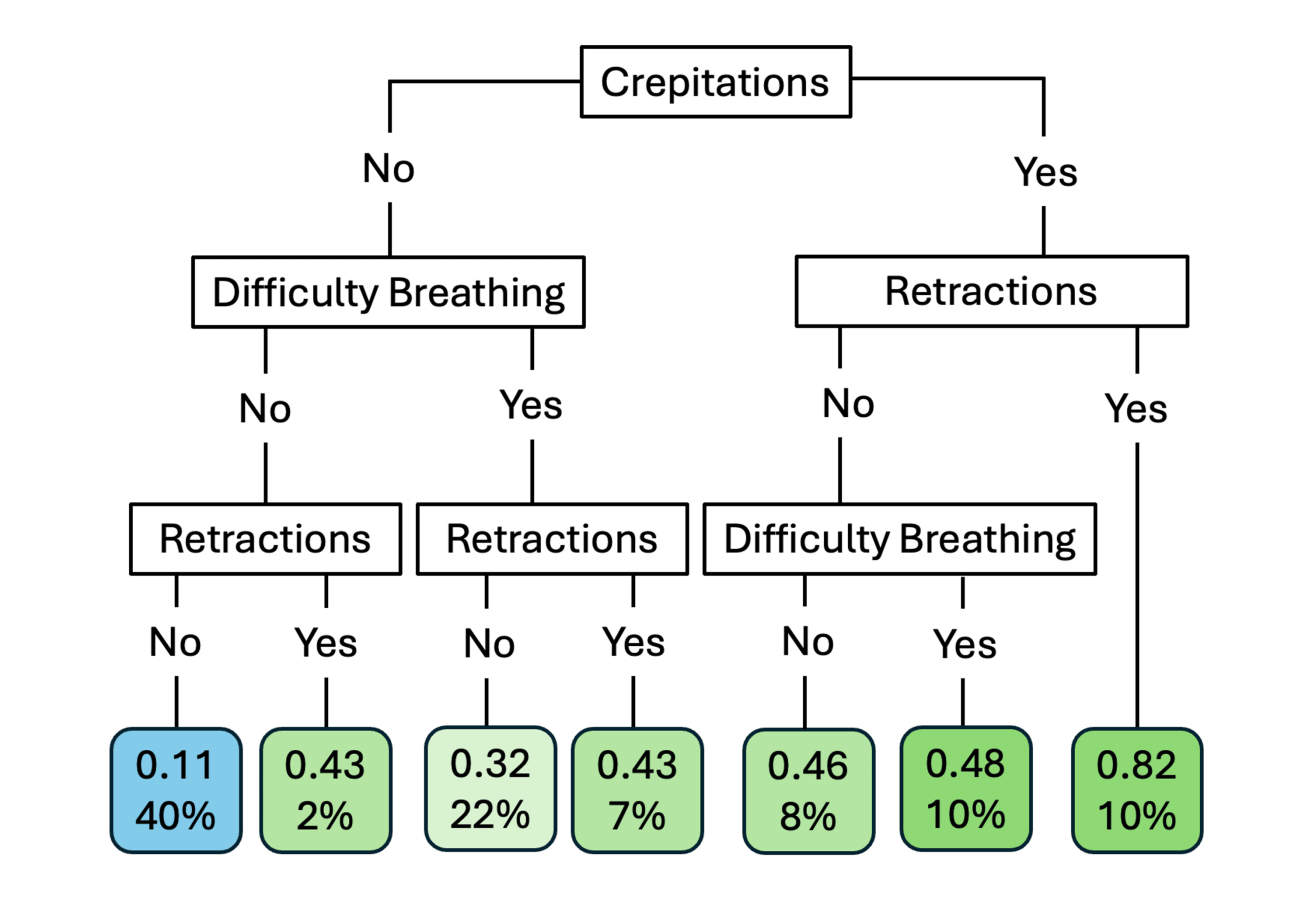
Prediction model of pneumonia based on clinical variables. The blue endpoint predicts no pneumonia and the green endpoints predict pneumonia with varying certainty. The top number for each endpoint is the predicted probability of having pneumonia, while percentages represent the percentage of patients that fit into that category.

**Figure 2:**
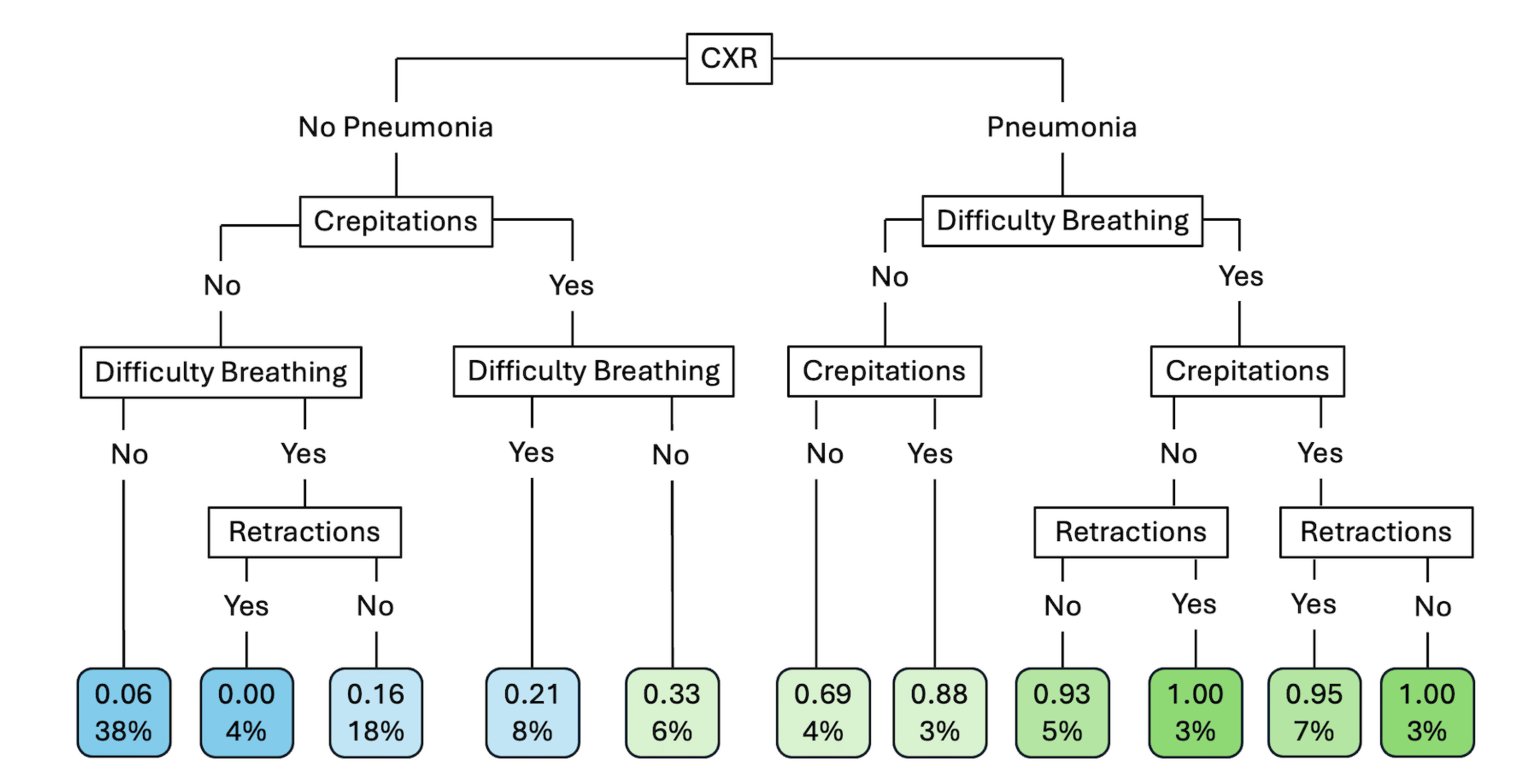
Prediction model of pneumonia adding chest X-ray with clinical characteristics. Blue endpoints predict no pneumonia and green endpoints predict pneumonia. The top number for each endpoint is the predicted probability of having pneumonia while percentages represent the percentage of patients that fit into that category.

Using recursive feature selection, three variables provided the best prediction for pneumonia: crepitations, retractions, and difficulty breathing. Using test data, the prediction model using these three clinical findings (Figure 1) provided a sensitivity of 74.2% and specificity of 38.5% with an F-score of 0.49. The best prediction for pneumonia was the presence of all three symptoms with a probability of 0.82; however, only 10% of the patients fit this category (Figure 1). The algorithm including chest X-ray (Figure 2) provided a sensitivity of 51.6% and specificity of 87.7% with a F-score of 0.58. If the chest X-ray was positive for pneumonia, patients had a high probability of pneumonia. However, probability for pneumonia still exists for patients with a negative chest X-ray, especially with crepitations (Figure 2).

The agreement between the clinician and expert reviewer for the presence or absence of pneumonia on lung ultrasound was excellent (k = 0.9).

## Discussion

This study is the first to evaluate individual clinical factors and combinations of factors that predict pneumonia in children under five using lung ultrasound as the gold standard. We found that no single clinical finding in isolation or combination of clinical findings provided enough accuracy to reliably diagnose pneumonia in children.

The combination of crepitations, difficulty breathing, and retractions provided a sensitivity of only 74%. These clinical findings are similar to the WHO criteria, which relies on tachypnea and chest indrawing or retractions [26]. Shrestha et al. found that tachypnea had a higher sensitivity but the addition of crepitations provided increased specificity [27]. This was also the case in our evaluation of individual clinical findings. However, when evaluating for the best combination, crepitations provided a better prediction model. The model found moderate to poor sensitivity at 74% and a specificity of only 38.5%. The model would not be reliable enough to rule in or rule out pneumonia leading to both under- and overdiagnosis and unnecessary antibiotics prescribed.

Overall, chest X-ray compared to lung ultrasound demonstrated moderate sensitivity and high specificity for pneumonia similar to previous studies [13,28]. The addition of a chest X-ray to the algorithm did not increase the sensitivity; however, it did improve the specificity and overall accuracy for pneumonia. This is consistent with expected outcomes as chest X-ray has a moderate sensitivity and high specificity for pneumonia, and the diagnostic test characteristics of the prediction model reflected the diagnostic accuracy of chest X-ray [13]. In addition to chest X-ray lacking sensitivity, the availability of chest X-ray also continues to be limited in rural and resource-limited settings.

The combination of clinical findings had limited sensitivity and accuracy for the diagnosis of pneumonia. While Chan et al. demonstrated a higher sensitivity (91.7%) with the PAFRI rule (a scoring system based on duration of fever, chills, nasal symptoms, abnormal chest exam, oxygen saturations <96% or tachypnea), the diagnosis was based on radiographic pneumonia as the gold standard [12]. Ramgopal et al. also utilized chest X-ray as the gold standard to develop a predictive model, finding age, fever duration, and decreased breath sounds to be predictors of pneumonia [29]. As both studies utilized chest X-ray as the gold standard, which has limited sensitivity, cases of pneumonia may have been missed limiting the accuracy of the prediction tools and study results.

In this study, no single clinical finding or combination of findings had adequate accuracy for predicting pneumonia in pediatric patients when compared to lung ultrasound as the standard. These results and the known high sensitivity and specificity of lung ultrasound for the diagnosis of pneumonia would suggest that lung ultrasound is the best way to rule in and rule out pneumonia [17,18,30]. Further study is needed to validate these findings.

This study has multiple limitations that limit the generalization of the study findings. The study was conducted in a single emergency department in urban Nepal, which may not be representative of all pediatric patients presenting in rural areas or other settings. Moreover, the accuracy of data collection for clinical findings is dependent on the healthcare provider’s skills and judgements, so variability in the assessments and documentation could introduce bias. Additional variability could be introduced in the chest X-ray reads, as they were read by only one radiologist. In order to reduce variability, the radiologists used a standardized methodology for diagnosing pneumonia. Even with the potential for variability, chest X-ray sensitivity and specificity were similar to previous studies. While we attempted to enroll all patients, without an electronic medical record, we were unable to track every patient and may have missed patients, which may lead to bias in sampling. We did enroll patients on every day of the week on all shifts, which provides a more representative sample. Moreover, our study did not use the high-frequency probe, which provides images with higher resolution in younger children. However, the curvilinear probe also has high sensitivity for diagnosis of pneumonia in children [30]. As curvilinear probes are often the only probe available in many low- and middle-income countries (LMICs), the study may be more applicable to LMICs than more resourced settings. Finally, our calculated sample size for this study was 400. Enrollment was stopped early due to the COVID-19 pandemic, with a total of 386 patients enrolled. While this is a limitation, we did reach the necessary 120 patients with pneumonia confirmed on lung ultrasound to obtain at least 10 outcome events in each independent variable analyzed.

## Conclusions

Using lung ultrasound as the gold standard, no single clinical finding or combination of clinical findings provided enough accuracy to reliably diagnose pneumonia in children under five years. Crepitations, difficulty breathing, and retractions provided the best combination to predict pneumonia, with a sensitivity similar to the WHO criteria. The addition of chest radiographs to these findings improved specificity but reduced sensitivity, which would result in more missed cases of pneumonia. Given the limitations of clinical exam and chest radiographs, lung ultrasound should be used for evaluating patients with concern for pneumonia. With the accuracy, availability, and ease of lung ultrasound, further studies implementing lung ultrasound for the diagnosis of pneumonia are needed in resource-limited settings.
